# PTEN regulates invasiveness in pancreatic neuroendocrine tumors through DUSP19-mediated VEGFR3 dephosphorylation

**DOI:** 10.1186/s12929-022-00875-2

**Published:** 2022-11-06

**Authors:** Tsung-Ming Chang, Pei-Yi Chu, Hui-You Lin, Kuo-Wei Huang, Wen-Chun Hung, Yan-Shen Shan, Li-Tzong Chen, Hui-Jen Tsai

**Affiliations:** 1 National Institute of Cancer Research, National Health Research Institutes, 1F No 367, Sheng-Li Road, Tainan, 70456 Taiwan; 2 Department of Medical Laboratory Science, College of Medical Science and Technology, I-Shou University, Kaohsiung, Taiwan; 3 Department of Pathology, Show Chwan Memorial Hospital, Changhua, Taiwan; 4 School of Medicine, College of Medicine, Fu Jen Catholic University, New Taipei City, Taiwan; 5 Department of Post-Baccalaureate Medicine, College of Medicine, National Chung Hsing University, Taichung, Taiwan; 6 Department of Surgery, National Cheng Kung University Hospital, College of Medicine, National Cheng Kung University, Tainan, Taiwan; 7 Institute of Clinical Medicine, College of Medicine, National Cheng Kung University, Tainan, Taiwan; 8 Department of Oncology, National Cheng Kung University Hospital, College of Medicine, National Cheng Kung University, Tainan, Taiwan; 9 Department of Internal Medicine, Kaohsiung Medical University Hospital, and Center for Cancer Research, Kaohsiung Medical University, Kaohsiung, Taiwan; 10 Department of Internal Medicine, Kaohsiung Medical University Hospital, Kaohsiung Medical University, Kaohsiung, Taiwan

**Keywords:** PTEN, VEGFR3, DUSP19, Pancreatic neuroendocrine tumor, Invasiveness

## Abstract

**Background:**

Phosphatase and tensin homolog (PTEN) is a tumor suppressor. Low PTEN expression has been observed in pancreatic neuroendocrine tumors (pNETs) and is associated with increased liver metastasis and poor survival. Vascular endothelial growth factor receptor 3 (VEGFR3) is a receptor tyrosine kinase and is usually activated by binding with vascular endothelial growth factor C (VEGFC). VEGFR3 has been demonstrated with lymphangiogenesis and cancer invasiveness. PTEN is also a phosphatase to dephosphorylate both lipid and protein substrates and VEGFR3 is hypothesized to be a substrate of PTEN. Dual-specificity phosphatase 19 (DUSP19) is an atypical DUSP and can interact with VEGFR3. In this study, we investigated the function of PTEN on regulation of pNET invasiveness and its association with VEGFR3 and DUSP19.

**Methods:**

PTEN was knocked down or overexpressed in pNET cells to evaluate its effect on invasiveness and its association with VEGFR3 phosphorylation. In vitro phosphatase assay was performed to identify the regulatory molecule on the regulation of VEGFR3 phosphorylation. In addition, immunoprecipitation, and immunofluorescence staining were performed to evaluate the molecule with direct interaction on VEGFR3 phosphorylation. The animal study was performed to validate the results of the in vitro study.

**Results:**

The invasion and migration capabilities of pNETs were enhanced by PTEN knockdown accompanied with increased VEGFR3 phosphorylation, ERK phosphorylation, and increased expression of epithelial–mesenchymal transition molecules in the cells. The enhanced invasion and migration abilities of pNET cells with PTEN knockdown were suppressed by addition of the VEGFR3 inhibitor MAZ51, but not by the VEGFR3-Fc chimeric protein to neutralize VEGFC. VEGFR3 phosphorylation is responsible for pNET cell invasiveness and is VEGFC-independent. However, an in vitro phosphatase assay failed to show VEGFR3 as a substrate of PTEN. In contrast, DUSP19 was transcriptionally upregulated by PTEN and was shown to dephosphorylate VEGFR3 via direct interaction with VEGFR3 by an in vitro phosphatase assay, immunoprecipitation, and immunofluorescence staining. Increased tumor invasion into peripheral tissues was validated in xenograft mouse model. Tumor invasion was suppressed by treatment with VEGFR3 or MEK inhibitors.

**Conclusions:**

PTEN regulates pNET invasiveness via DUSP19-mediated VEGFR3 dephosphorylation. VEGFR3 and DUSP19 are potential therapeutic targets for pNET treatment.

**Supplementary Information:**

The online version contains supplementary material available at 10.1186/s12929-022-00875-2.

## Background

Pancreatic neuroendocrine tumors (pNETs) are neoplasms of pancreatic origin with significant neuroendocrine differentiation and expression of neuroendocrine markers [1]. pNETs account for approximately 3% of all pancreatic neoplasms in Taiwan, and the incidence of pNETs has significantly increased worldwide in recent decades [2–4]. The prognosis of pNETs is significantly associated with stage. The overall survival time of the patients with pNETs with regional and distant metastases is significantly shorter than that of the patients with pNETs at a localized stage [3]. Metastasis is a great challenge for the clinical management of the patients. It is well known that the metastatic ability of cancer cells is strongly associated with cell motility, such as cell migration and invasion [5]. Therefore, it is necessary to understand the molecular mechanisms of cell invasiveness in pNETs to prevent and manage metastatic disease.

Phosphatase and tensin homolog (PTEN) is a phosphatidylinositol-3,4,5-trisphosphate 3-phosphatase that acts as a tumor suppressor by inhibiting the AKT signaling pathway and regulates cell behaviors, including cell growth, motility, and invasiveness [6]. Low PTEN expression is associated with a higher proportion of liver metastases and shorter disease-free survival/progression-free survival and overall survival times in the patients with pNET [7, 8]. We previously showed that loss of PTEN promotes the proliferation of pNETs in vitro via upregulation of the AKT/mTOR/c-Myc axis [9]. Whether PTEN affects cell invasiveness in pNETs is unclear. Vascular endothelial growth factor receptor 3 (VEGFR3) is a member of the class III receptor tyrosine kinase family that contains seven immunoglobulin-like loops [10]. VEGFR3, which is predominantly expressed in lymphatic vessels and has been identified as a marker for lymphatic endothelial cells. The binding of vascular endothelial growth factor (VEGF) C and VEGFD to VEGFR3, along with the activation of downstream signaling, is essential for lymphangiogenesis [11, 12]. VEGFR3 is also expressed in various tumor cells and plays a role in tumor growth and metastasis in many cancer types [13, 14]. However, the functional role of VEGFR3 in pancreatic neuroendocrine tumorigenesis has not been fully explored. In this study, we investigated the roles of PTEN and VEGFR3 in the invasiveness of pNETs, and delineated the molecular mechanisms by which PTEN regulates VEGFR3 phosphorylation in pNETs.

## Materials and methods

### Cell lines, plasmids, and reagents

We purchased human QGP-1 cells from the Japanese Collection of Research Bioresources (*JCRB*; Tokyo, Japan), and murine NIT-1 cells were obtained from the Bioresource Collection and Research Center (BCRC; Hsinchu, Taiwan). QGP-1 cells were cultured in RPMI-1640 medium (HyClone, South Logan, UT, USA) containing 10% fetal bovine serum (FBS) and antibiotics. NIT-1 cells were cultured in F12-Kaighn’s medium (Gibco, Grand Island, NY, USA) containing 10% FBS and antibiotics. The cell lines were used in this study at or before passage 15. Human wild-type and mutant PTEN (C124S, G129E, and Y138L) [6], and human dual-specificity protein phosphatase 19 (DUSP19) expression plasmids were purchased from Addgene (Cambridge, MA, USA). shRNAs targeting Luc (shLuc), PTEN (shPTEN), VEGFR3 (shVEGFR3), and DUSP19 (shDUSP19) were obtained from the National RNAi Core Facility of Academic Sinica (Taipei, Taiwan). shLuc was used as a control for gene knockdown. Recombinant human VEGFR3-Fc chimeric protein and recombinant human PTEN protein were purchased from R&D Systems (Minneapolis, MN, USA). The VEGFR3 kinase inhibitor, MAZ51, was purchased from Calbiochem (San Diego, CA, USA). Trametinib (GSK1120212) was purchased from AdooQ Biosciences (Irvine, CA, USA). Biotinylated VEGFR3 phosphopeptide was custom-made by Mission Biotech (Taipei, Taiwan). Recombinant human DUSP19 protein was purchased from Origene (Rockville, MD, USA).

### Plasmid transfection and lentiviral transduction

Knockdown of PTEN, VEGFR3, and DUSP19 by shRNA in the indicated cell lines was conducted using a lentiviral transduction system according to the instructions of the National RNAi Core Facility of Academic Sinica. Overexpression of human wild-type PTEN, mutant PTEN (C124S, G129E, and Y138L), and human DUSP19 was performed using the Lipofectamine 2000 transfection reagent (Invitrogen, Carlsbad, CA, USA).

### RNA extraction and RT-PCR

Total RNA was isolated from control, PTEN knocked down or PTEN overexpressed QGP-1 cells by using a total RNA mini kit (Geneaid, New Taipei City, Taiwan). A high-capacity cDNA reverse transcription kit (Applied Biosystems Inc, Waltham, MA, USA) was used to perform reverse transcription according to the manufacturer’s protocol. DUSP19 expression was examined by using SYBR green PCR master mix, and GAPDH was used as an internal control to check the efficiency of cDNA synthesis and PCR amplification. The primers used were: DUSP19-forward, 5′-CCAGTGCTTTTTCTTTGGTG-3′; DUSP19-reverse, 5′-TTGCTTTCTTTGCCCTCTTG-3′; GAPDH-forward, 5′-ACGTGATGCAGAACCACCTACTG-3′; and GAPDH-reverse, 5′-ACGACGGCTGCAAAAGTGGCG-3′. The PCR products were separated on a 3% 0.5× Tris–acetate-EDTA agarose gel and visualized under a UVP Biospectrum image system (Upland, CA, USA).

### Western blot analysis

Whole cell lysates were harvested using a lysis buffer. Equal amounts of protein were subjected to SDS-PAGE and transferred onto PVDF membranes. The membranes were incubated with different primary antibodies, including anti-VEGFR3, anti-PTEN, anti-GAPDH (Santa Cruz Biotechnology, Dallas, TX, USA), anti-phospho-VEGFR3 (Cell Applications, San Diego, CA, USA), anti-SLUG, anti-vimentin, anti-p38, anti-phospho-p38, anti-ERK, anti-phospho-ERK, anti-phospho-JNK, anti-AKT, anti-phospho-AKT (Cell Signaling Technology, Danvers, MA, USA), anti-E-cadherin (BD Biosciences, Franklin Lakes, NJ, USA), and anti-DUSP19 (GeneTex, Irvine, CA, USA). The immunocomplexes were detected by probing with anti-mouse or -rabbit IgG conjugated with horseradish peroxidase (Jackson ImmunoResearch, West Grove, PA, USA). Immunoreactive bands were detected on membranes by adding an enhanced chemiluminescence reagent (PerkinElmer Western Lightning Plus-ECL, PerkinElmer, Waltham, MA, USA). Bands were visualized using a UVP BioSpectrum imaging system (Upland, CA, USA). The details of western blot analysis are described in our previous paper [15].

### In vitro cell invasion and migration assays

The in vitro invasion assay was performed using 24-well Transwell units with polycarbonate filters (pore size, 8 μm) coated with Matrigel (BD Biosciences) on the upper surface. The lower compartment of the Transwell unit was filled with a medium containing 10% FBS. A total of 1 × 10^4^ cells were seeded in the upper compartment of the Transwell unit, which allowed these cells to invade. Non-invading cells on the upper surface of the membrane were removed after 24 h using a cotton swab. The invading cells on the bottom surface of the membrane were fixed with formaldehyde and stained with the Giemsa solution. Cells were counted under a microscope. For the migration assay, the polycarbonate filters were not coated with matrigel. The other procedures were the same as those used for the invasion assay.

### Immunoprecipitation and immunofluorescence staining

For immunoprecipitation, cell lysates containing 1000 μg of cellular protein were incubated with anti-VEGFR3 antibody (sc-514825, Santa Cruz Biotechnology), anti-DUSP19 (GeneTex) or mouse IgG (sc-2762, Santa Cruz Biotechnology) overnight at 4 °C, and the immunocomplexes were collected with immunoprecipitation reagents. The collected proteins were released by boiling in SDS-PAGE loading buffer and subjected to SDS-PAGE. Western blotting was performed by probing the membranes with anti-VEGFR3 anti-mouse and anti-DUSP19 (GeneTex) anti-rabbit antibodies to detect interactions between VEGFR3 and DUSP19. For immunofluorescence staining, cells were cultured on coverslips, fixed with 4% formaldehyde for 15 min at room temperature, and washed with phosphate-buffered saline (PBS). The cells were permeabilized with 0.1% Triton X-100 for 10 min and incubated with 1% bovine serum albumin to block non-specific binding. Anti-VEGFR3 and anti-DUSP19 antibodies were applied to coverslips and incubated overnight at 4 °C. After extensive washing with PBS, Alexa Fluor 488-conjugated anti-rabbit IgG and Alexa Fluor 594-conjugated anti-mouse IgG (Invitrogen) were added to the coverslips and incubated at room temperature for 1 h. The coverslips were washed twice with PBS and then added with DAPI working solution for 5 min incubation at room temperature with protection from light. The coverslips were then washed twice with PBS and placed in mounting solution. The locations of the fluorescence signals were observed using a fluorescence microscope (LEICA DMI4000 B, Leica Microsystems, Wetzlar, Germany). Immunofluorescence images were also obtained and reconstituted by LEICA DMi8 microscope and Leica Application Suite X (LAS X) software (*Leica* Microsystems) to produce the XY section and XZ section images.

### In vitro phosphatase assay

Biotinylated VEGFR3 phosphopeptide was added to the streptavidin-coated 96-well plates and incubated for 2 h at room temperature. The excess (biotinylated) phosphopeptide was then removed using wash buffer (PBS containing 0.05% Tween 20). Recombinant PTEN or DUSP19 was added to each well and the plate was incubated for 1 h at 37 °C. Recombinant PTEN or DUSP19 was removed using wash buffer and the primary antibody (anti-phospho-VEGFR3) was added to each well of the plate and incubated for 1 h at room temperature. The primary antibody was removed with wash buffer and horseradish peroxidase (HRP)-conjugated secondary antibodies (Biolegend, San Diego, CA, USA) was added to each well and incubated for 1 h at room temperature. Finally, each well of the plate was washed three times with wash buffer and incubated with tetramethylbenzidine (TMB) substrate solution (Clinical Science Products, Mansfield, MA, USA) for 15 min at room temperature prior to measuring the absorbance of each well at 405 nm (Sunrise absorbance reader, TECAN, Männedorf, Switzerland).

### Animal study

Male NOD-SCID mice (6 to 8-week old) were obtained from LASCO (Taipei, Taiwan). Animals were housed under specific pathogen–free conditions according to the guidelines of the Animal Care Committee of the National Health Research Institutes (NHRI), Taiwan. The mice were provided with free access to a standard sterilized laboratory diet and water. QGP-1 cells transduced with shLuc or shPTEN plasmid (1.0 × 10^7^ cells/mouse) were inoculated into the mice by injection into the subcutaneous space in the flank. After approximately 2 weeks, the tumors had grown to a volume of approximately 150–200 mm^3^. There were 10 mice in the shLuc control group (QGP-1/shLuc) and 30 in the shPTEN group. Thirty mice, inoculated with QGP-1/shPTEN cells, were randomly divided into three groups. Mice in one group (shPTEN/control) were orally administered with PBS (control group) (n = 10). Another 10 mice were treated with 1 mg/kg of trametinib (shPTEN + trametinib group). The remaining ten mice were treated intraperitoneally with 8 mg/kg MAZ51 (shPTEN + MAZ51 group). Tumor volumes were measured twice a week until sacrifice. Tumor volumes were calculated using the formula V = length (mm) × width^2^ (mm) × (π/6) [16]. The animals were sacrificed by CO_2_ exposure in their home cages, and the tumors were excised for further analysis.

### Immunohistochemical (IHC) staining

The animal tissues were fixed in 10% neutral-buffered formalin and then underwent tissue processing, embedding in paraffin wax to make paraffin blocks. Sections (4 µm) sliced using a microtome from paraffin-embedded tissue blocks were in 10 mM Tris-buffered saline containing 0.5% Tween 20, pH 7.6, rehydrated through sequential dilutions of alcohol, and washed in PBS. The slides stained with anti-SLUG primary antibody (1:100; sc-166476, Santa Cruz Biotechnology) were performed on the BOND-MAX Automated Immunohistochemistry Vision Biosystem (Leica Microsystem) using onboard heat induced antigen retrieval and a Leica Bond Polymer Refine Detection system (Leica Biosystems, Wetzlar, Germany). Diaminobenzidine (DAB) was used as the chromogen (Leica Biosystems) in all the immunostainings. To prepare a negative control, IHC was performed without the primary antibody.

The invasion status was scored by the extent of tumor invasion to the surrounding tissues. Confinement of the tumor to the original area was scored as 0, focal invasion into the peripheral fibrofatty tissue as 1, multifocal invasion into the peripheral fibrofatty tissue as 2, and invasion into the muscular bundles as 3. To analyze SLUG expression, a semiquantitative assessment of the percentage of positively stained carcinoma cells (from 0 to 100%) along with the staining intensity (graded as 0, no staining; 1, weak staining; 2, moderate staining; and 3, strong staining) was used. Finally, a board-certified pathologist blinded to the specimen information independently assessed the SLUG expression.

### A search of RNA sequencing database for identifying correlation of *PTEN* and *DUSP19* genes in pNET tumors

RNA sequencing data of 33 pNETs were collected from the GEO GSE118014 database (http://www.ncbi.nlm.nih.gov/geo/). Gene expression was examined by Illumina HiSeq 2000. Raw data were obtained to analyze correlation between *PTEN* and *DUSP19* genes [17].

### Statistical analysis

SAS statistical software (version 9.4, SAS Institute Inc., Cary, NC, USA) was used to perform statistical analyses. Differences in the relative migration rate, invasion rate, and phosphatase activity between pNET cells under the indicated conditions were analyzed using the Wilcoxon rank-sum test. The difference in tumor invasion between the inoculated tumors under the indicated conditions was analyzed using the Wilcoxon rank-sum test. Differences in SLUG expression between inoculated tumors under the indicated conditions were analyzed using Fisher’s exact test. Spearman’s rank-order correlation was calculated and correlation coefficiency (ρ) was expressed to correlate mRNA expression level of PTEN and DUSP19 in pNET tumors from RNA sequencing database. A two-sided *P* value of less than 0.05 indicated significance.

## Results

### PTEN loss enhances the invasiveness of pNET cells and increases VEGFR3 phosphorylation

**Fig. 1:**
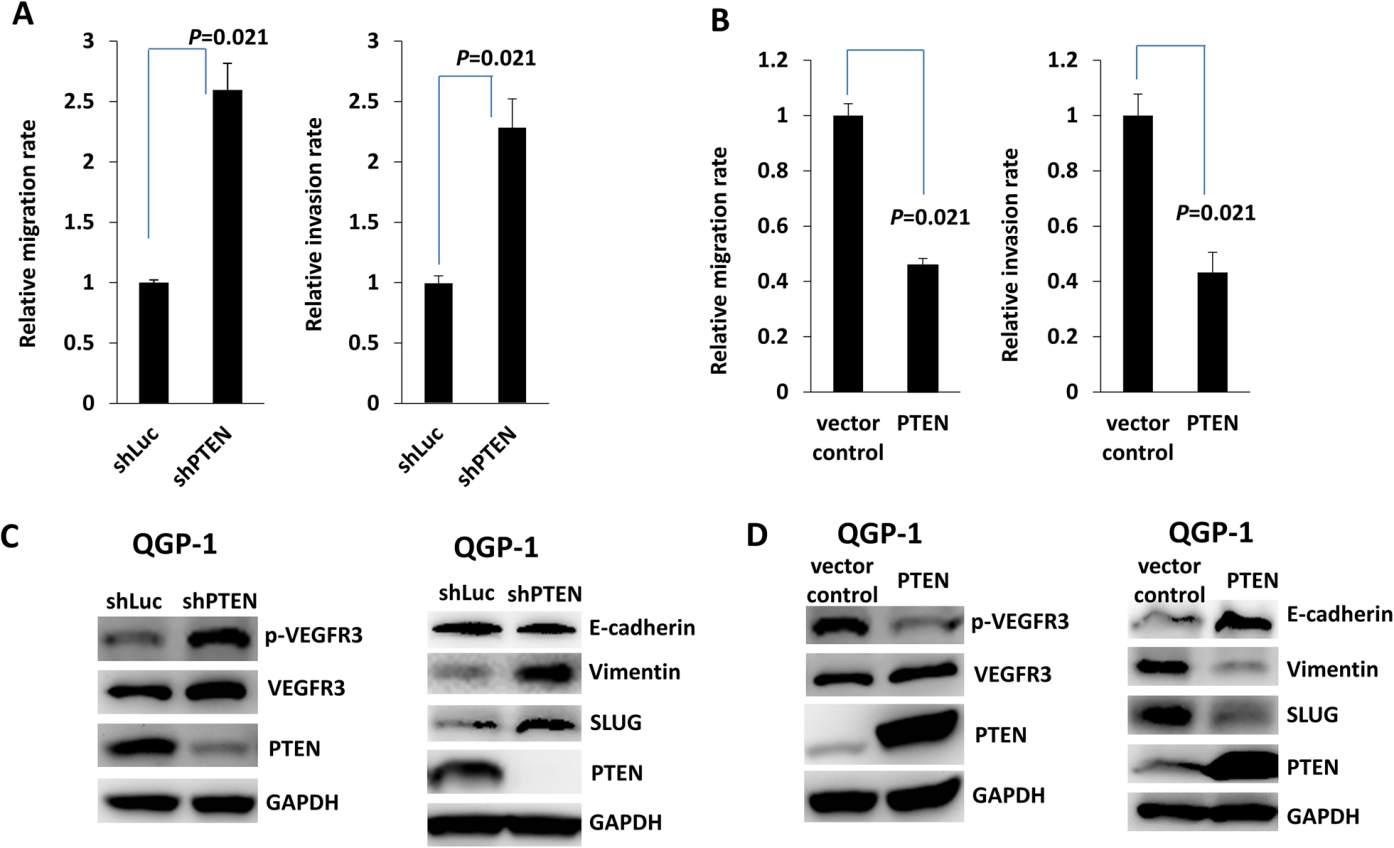
The migration and invasion abilities of QGP-1 cells are negatively regulated by PTEN. **A** The relative migration (*P* = 0.021, Wilcoxon rank-sum test) and invasion (*P* = 0.021, Wilcoxon rank-sum test) abilities of QGP-1 cells with and without knockdown of PTEN. **B** The relative migration (*P* = 0.021, Wilcoxon rank-sum test) and invasion (*P* = 0.021, Wilcoxon rank-sum test) abilities of QGP-1 cells with and without overexpression of PTEN. **C** The protein levels of VEGFR3, phosphorylated VEGFR3, E-cadherin, vimentin and SLUG in QGP-1 cells with and without PTEN knockdown. **D** The protein levels of VEGFR3, phosphorylated VEGFR3, E-cadherin, vimentin and SLUG in QGP-1 cells with and without overexpression of PTEN

To understand whether PTEN expression correlates with the invasiveness of pNETs, we knocked down and overexpressed PTEN in a human pNET cell line, QGP-1, and evaluated the invasion and migration abilities of QGP-1 cells. Figure 1A shows that migration and invasion were significantly enhanced in QGP-1 cells with PTEN knockdown. In contrast, migration and invasion were significantly suppressed in QGP-1 cells overexpressing PTEN (Fig. 1B). We evaluated VEGFR3 expression in QGP-1 cells with and without PTEN knockdown or overexpression. Figure 1C shows that VEGFR3 phosphorylation significantly increased in QGP-1 cells upon PTEN knockdown. The molecules associated with epithelial–mesenchymal transition (EMT) were also evaluated. Decreased protein expression of E-cadherin and increased protein expression of vimentin and SLUG were noted in QGP-1 cells with PTEN knockdown (Fig. 1C). Figure 1D shows that VEGFR3 phosphorylation was significantly reduced in the QGP-1 cells overexpressing PTEN. The protein expression of E-cadherin increased, whereas that of vimentin and SLUG decreased in QGP-1 cells overexpressing PTEN. The enhancement of migration and invasion and the increase in VEGFR3 phosphorylation in pNET cells was also demonstrated in a murine pNET cell line, NIT-1, with PTEN knockdown accompanied by associated changes in EMT molecules (Additional file 1: Fig. S1). These data demonstrated that PTEN regulates cell invasiveness and VEGFR3 phosphorylation in pNETs.

### PTEN-regulated VEGFR3 phosphorylation is responsible for pNET cell invasiveness in a VEGFC-independent manner

**Fig. 2:**
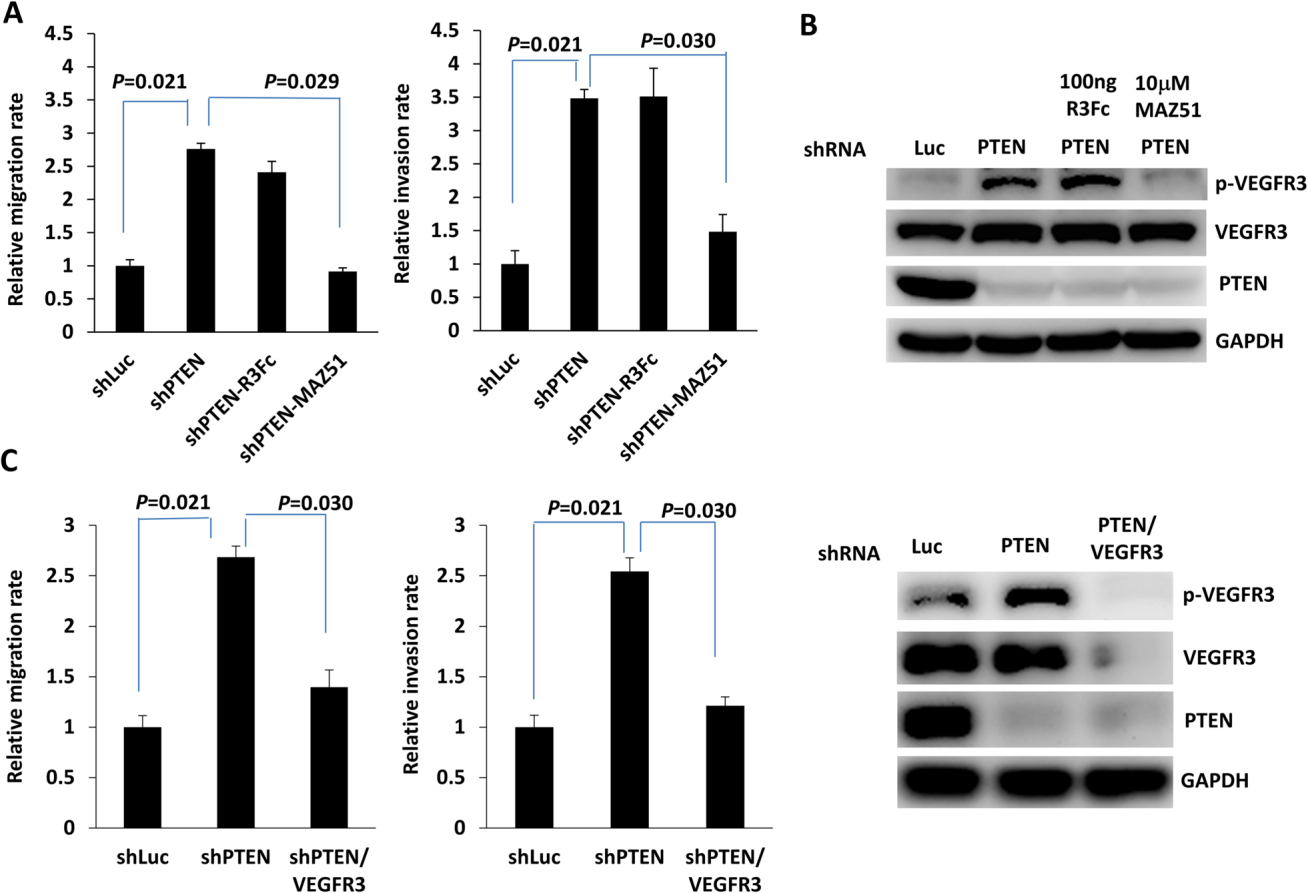
VEGFR3 phosphorylation is responsible for pNET cell invasiveness in a VEGFC-independent manner. **A** The relative migration and invasion abilities of QGP-1 cells with (shPTEN) and without (shLuc) knockdown of PTEN and treated with the VEGFR3-Fc chimera protein (shPTEN-R3Fc) or the VEGFR3 inhibitor MAZ51 (shPTEN-MAZ51). Migration: shLuc vs. shPTEN, *P* = 0.021; shPTEN vs. shPTEN-MAZ51, *P* = 0.030; Wilcoxon rank-sum test. Invasion: shLuc vs. shPTEN, *P* = 0.021; shPTEN vs. shPTEN-MAZ51, *P* = 0.029; Wilcoxon rank-sum test. **B** The protein level of phosphorylated VEGFR3 in QGP-1 cells with and without knockdown of PTEN and treated with the VEGFR3-Fc chimera protein or the VEGFR3 inhibitor MAZ51. **C** The relative migration and invasion abilities of QGP-1 cells with (shPTEN) and without (shLuc) knockdown of PTEN and with (shPTEN/VEGFR3) or without knockdown of VEGFR3. Migration: shPTEN vs. shPTEN/VEGFR3, *P* = 0.030; Wilcoxon rank-sum test. Invasion: shPTEN vs. shPTEN/VEGFR3, *P* = 0.030; Wilcoxon rank-sum test. The protein level of phosphorylated VEGFR3 in QGP-1 cells with and without knockdown of PTEN and with or without knockdown of VEGFR3 was shown in right panel

**Fig. 3:**
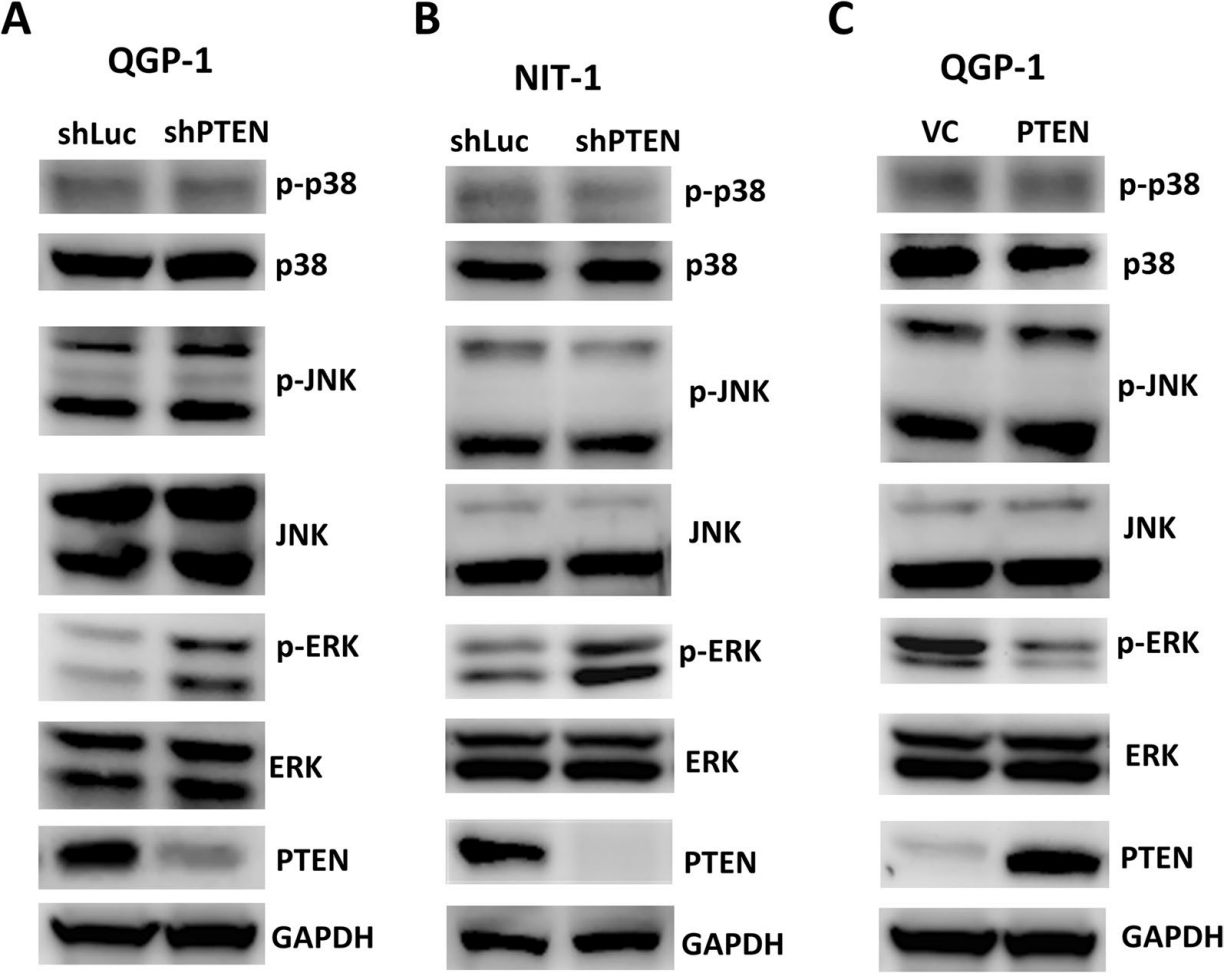
ERK is the downstream effector of VEGFR3 upregulated in pNET cells with PTEN loss. **A** The protein levels of p38, JNK, ERK and their phosphorylated forms in QGP-1 cells with and without PTEN knockdown. **B** The protein levels of p38, JNK, ERK and their phosphorylated forms in NIT-1 cells with and without PTEN knockdown. **C** The protein levels of p38, JNK, ERK and their phosphorylated forms in QGP-1 cells with and without overexpression of PTEN

**Fig. 4:**
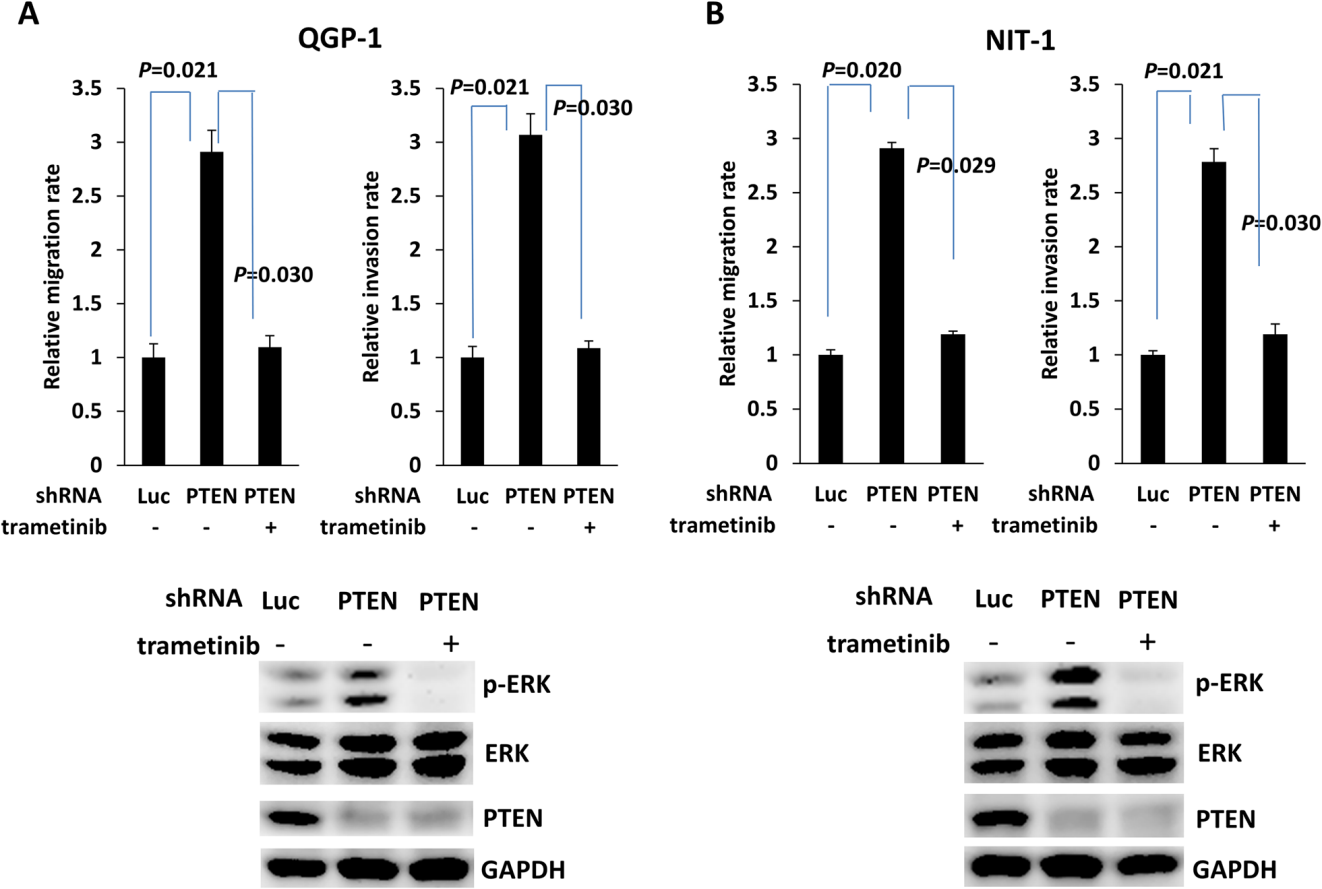
Trametinib can suppress the increases in the migration and invasion capabilities of pNETs induced by PTEN loss. **A** The relative migration and invasion abilities of and ERK phosphorylation status in QGP-1 cells with (shPTEN) and without (shLuc) knockdown of PTEN and with (shPTEN + trametinib) and without trametinib treatment. Migration: shPTEN vs. shPTEN + trametinib, *P* = 0.030; Wilcoxon rank-sum test. Invasion: shPTEN vs. shPTEN + trametinib, *P* = 0.030; Wilcoxon rank-sum test. **B** The relative migration and invasion abilities of and ERK phosphorylation status in NIT-1 cells with (shPTEN) and without (shLuc) knockdown of PTEN and with (shPTEN + trametinib) and without trametinib treatment. Migration: shPTEN vs. shPTEN + trametinib, *P* = 0.029; Wilcoxon rank-sum test. Invasion: shPTEN vs. shPTEN + trametinib, *P* = 0.030; Wilcoxon rank-sum test

To confirm whether PTEN-regulated VEGFR3 phosphorylation is associated with the invasiveness of pNETs, we added the VEGFR3 kinase inhibitor MAZ51 to the culture medium of QGP-1 cells with PTEN knockdown to measure the migration and invasion capabilities of the cells (Fig. 2A). The increase in migration and invasion capabilities of QGP-1 cells upon PTEN knockdown was inhibited by the addition of MAZ51. VEGFC is a major ligand for VEGFR3, which induces receptor phosphorylation and activation of its downstream signaling pathway in human tissues and cancers [11, 12]. We previously showed that PTEN loss induces upregulation of c-Myc and that c-Myc promotes VEGFC upregulation and lymphangiogenesis in pNET cells [9, 18]. To evaluate whether upregulation of VEGFR3 expression in QGP-1 cells with PTEN knockdown is associated with enhanced VEGFC binding, we added a VEGFR3-Fc chimeric protein, R3Fc, to the culture medium of QGP-1 cells with PTEN knockdown. No suppressive effect on cell migration or invasion was observed in culture with the addition of R3Fc (Fig. 2A). Figure 2B shows that the increase in VEGFR3 phosphorylation in QGP-1 cells with PTEN knockdown was reduced by the addition of MAZ51 but not R3Fc. A similar result was observed in the murine pNET cell line, NIT-1 (Additional file 2: Fig. S2). In addition, we knocked down VEGFR3 in QGP-1 cells with PTEN knockdown and found that the enhanced migration and invasion capabilities of QGP-1 cells with PTEN knockdown was significantly suppressed by VEGFR3 knockdown (Fig. 2C). We evaluated the downstream signals of VEGFR3 associated with invasion and migration, including p38, JNK, and ERK, in QGP-1 cells with PTEN knockdown or overexpression, respectively. Only ERK phosphorylation significantly increased in QGP-1 cells after PTEN knockdown (Fig. 3A). A similar result was observed in NIT-1 (Fig. 3B). In contrast, ERK phosphorylation was significantly reduced in QGP-1 cells overexpressing PTEN (Fig. 3C). We added a MEK inhibitor, trametinib, to the culture medium of QGP-1 cells with PTEN knockdown. Figure 4A shows that the enhanced migration and invasion capabilities of QGP-1 cells with PTEN knockdown were significantly suppressed by the addition of trametinib, accompanied by a decrease in the phosphorylated ERK level in cells with PTEN knockdown. A similar result was observed in NIT-1 (Fig. 4B). These results indicate that PTEN regulates VEGFR3 phosphorylation independent of VEGFC, and that PTEN-regulated VEGFR3 phosphorylation is responsible for the invasiveness of pNET cells. Blockade of VEGFR3 or ERK can reverse the increase in the invasiveness of pNET cells with PTEN loss.

### PTEN transcriptionally regulates DUSP19 to dephosphorylate VEGFR3 in pNETs

**Fig. 5:**
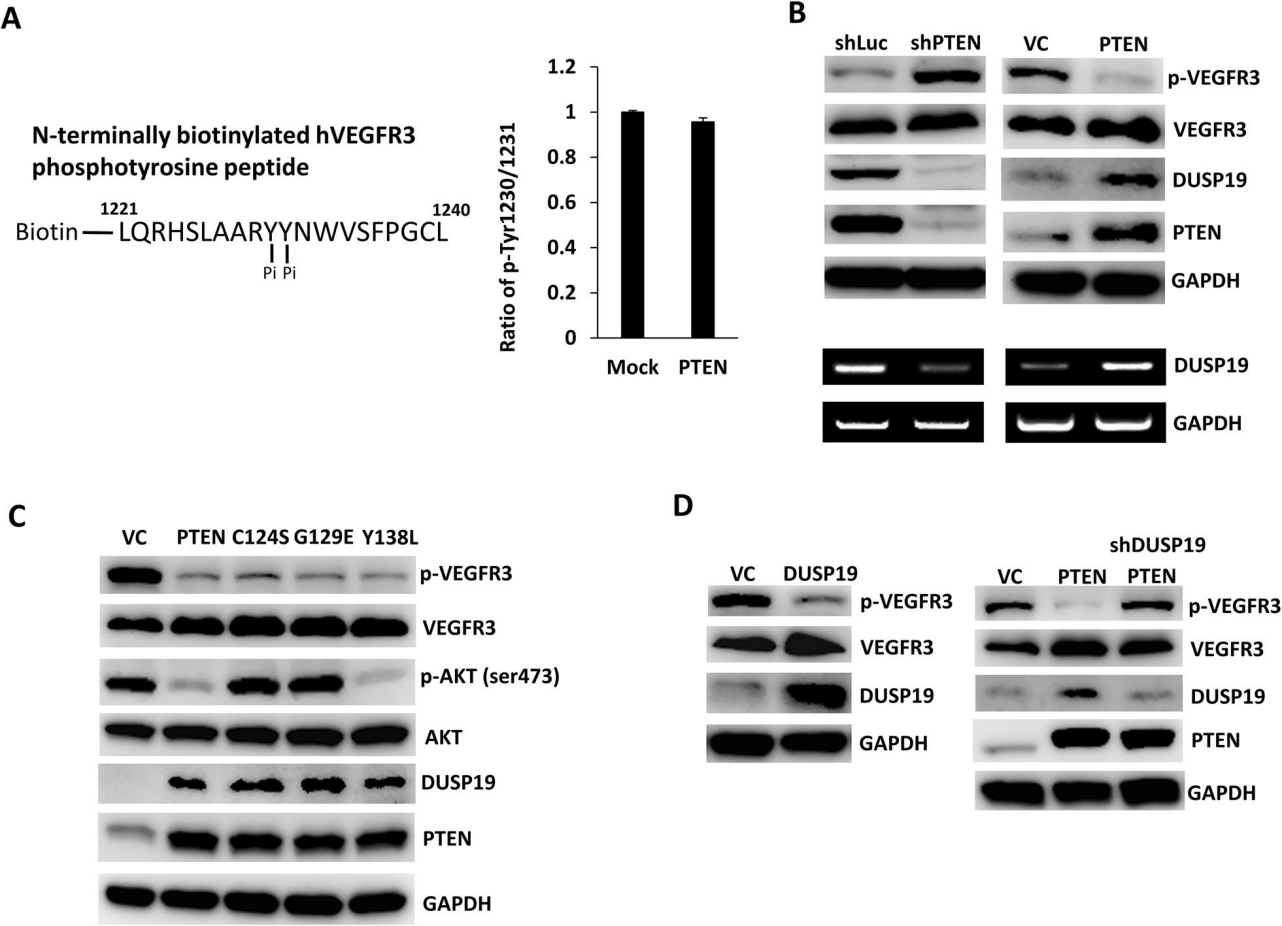
PTEN transcriptionally regulates DUSP19 to dephosphorylate VEGFR3 in pNETs. **A** The phospho-signal of the phospho-VEGFR3 peptide was not changed by the addition of recombinant PTEN. **B** The protein expression levels of phosphorylated VEGFR3, VEGFR3, DUSP19, and PTEN in QGP-1 cells with PTEN knockdown or overexpression (upper panel). The mRNA expression levels of DUSP19 in QGP-1 cells with PTEN knockdown or overexpression (lower panel). **C** The protein levels of phosphorylated VEGFR3, VEGFR3, phosphorylated AKT, AKT, DUSP19, and PTEN in QGP-1 cells overexpressing wild-type, lipid phosphatase-deficient (G129E), protein phosphatase-deficient (Y138L) and both lipid and protein phosphatase-deficient (C124S) PTEN. **D** The protein level of phosphorylated VEGFR3 in QGP-1 cells with and without DUSP19 overexpression (left). The protein level of phosphorylated VEGFR3 in QGP-1 cells with and without PTEN overexpression and in QGP-1 cells with PTEN overexpression and DUSP19 knockdown (right)

We then attempted to delineate the molecular mechanism through which PTEN regulatesVEGFR3 phosphorylation. Although PTEN principally has lipid phosphatase activity, it also has a protein tyrosine phosphatase catalytic domain [6, 19]. To evaluate whether PTEN directly dephosphorylates VEGFR3, we performed an in vitro phosphatase assay by incubating recombinant human PTEN with biotinylated VEGFR3 phosphopeptide. The phospho-signal of the phospho-VEGFR3 peptide was unchanged by addition of recombinant PTEN (Fig. 5A). This result indicated that PTEN does not regulate VEGFR3 phosphorylation through direct interaction with VEGFR3. We also investigated other potential mediators of VEGFR3 phosphorylation. A global analysis of the receptor tyrosine kinase–protein phosphatase interactome showed that VEGFR3 interacts with DUSP19 [20]. This finding suggests that DUSP19 may be a critical mediator of VEGFR3 dephosphorylation by PTEN. DUSPs are heterogeneous protein phosphatases that can dephosphorylate tyrosine and serine/threonine residues on the same substrate. DUSP19 is classified as an atypical DUSP [21]. We evaluated the protein and mRNA expression level of DUSP19 in QGP-1 cells with PTEN knockdown or overexpression. Figure 5B shows that the protein (upper panel) and mRNA (lower panel) level of DUSP19 was significantly reduced in QGP-1 cells with PTEN knockdown. In contrast, the protein and mRNA level of DUSP19 was significantly increased in QGP-1 cells overexpressing PTEN. The protein level of DUSP19 was positively correlated with that of PTEN but negatively correlated with VEGFR3 phosphorylation. In addition, Fig. 5C shows that the protein level of DUSP19 increased in QGP-1 cells overexpressing wild-type, lipid phosphatase-deficient (G129E), protein phosphatase-deficient (Y138L), and both lipid and protein phosphatase-deficient (C124S) PTEN [6]. This result indicated that the mechanism by which PTEN upregulates DUSP19 expression is independent of phosphatase activity. We overexpressed DUSP19 in QGP-1 cells, and reduced the phosphorylation of VEGFR3 in QGP-1 cells was noted (Fig. 5D). Furthermore, we knocked down DUSP19 expression in the QGP-1 cells overexpressing PTEN. Knockdown of DUSP19 restored VEGFR3 phosphorylation in QGP-1 cells overexpressing PTEN, which led to dephosphorylation of VEGFR3 (Fig. 5D). This result demonstrated that VEGFR3 phosphorylation is regulated by DUSP19. These results supported our hypothesis that PTEN transcriptionally upregulates DUSP19 expression to dephosphorylate VEGFR3 in pNET.

### DUSP19 dephosphorylates VEGFR3 via a direct receptor tyrosine kinase–protein phosphatase interaction

**Fig. 6:**
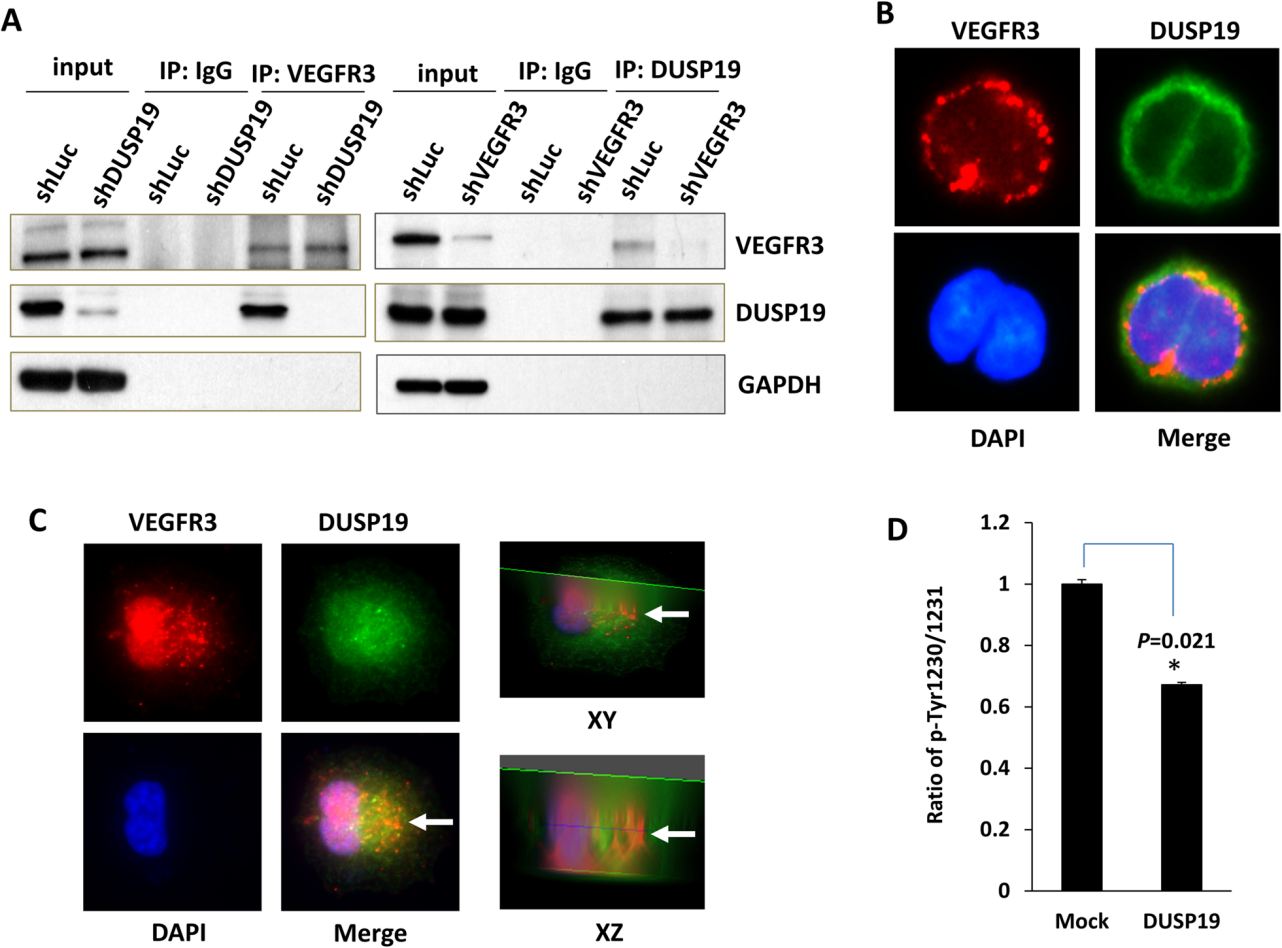
DUSP19 dephosphorylates VEGFR3 via a direct receptor tyrosine kinase–protein phosphatase interaction. **A** Western blot analysis of the VEGFR3 immunoprecipitates showed that DUSP19 was present in the immunocomplex from QGP-1 cells with knockdown of vector control (shLuc) but not present in the immunocomplex from QGP-1 cells with DUSP19 knockdown (left panel). Western blot analysis of the DUSP19 immunoprecipitates showed that VEGFR3 was present in the immunocomplex from QGP-1 cells with knockdown of vector control (shLuc) but not present in the immunocomplex from QGP-1 cells with VEGFR3 knockdown (right panel). Immunoprecipitation with IgG was used as negative control. **B** VEGFR3 and DUSP19 were shown to be colocalized on the cell membrane of QGP-1 cells by immunofluorescence staining. **C** Confocal microscope images of XY and XZ sections. **D** The phospho-signal of the phospho-VEGFR3 peptide was reduced by the addition of recombinant DUSP19 (*P* = 0.021, Wilcoxon rank-sum test)

To confirm the receptor tyrosine kinase–protein phosphatase interaction of VEGFR3 and DUSP19, an anti-VEGFR3 antibody was used to pull down VEGFR3-associated protein complexes in QGP-1 cells with or without knockdown of DUSP19. DUSP19 was present in the protein complex with VEGFR3 from QGP-1 cells with knockdown of vector control (shLuc) but not present in the protein complex with VEGFR3 from QGP-1 cells with DUSP19 knockdown (shDUSP19). The mouse IgG was used as a negative control for pull down assay (Fig. 6A, left panel). On the other hand, an anti-DUSP19 antibody was used to pull down DUSP19-associated protein complexes in QGP-1 cells with or without knockdown of VEGFR3. VEGFR3 was present in the protein complex with DUSP19 from QGP-1 cells with knockdown of vector control (shLuc) but not present in the protein complex with DUSP19 from QGP-1 cells with VEGFR3 knockdown (shVEGFR3) (Fig. 6A, right panel). Immunofluorescence staining of QGP-1 cells showed that VEGFR3 and DUSP19 were colocalized on the cell membrane (Fig. 6B). Furthermore, z stack image of confocal microscope also showed the co-localization of VEGFR3 and DUSP19 (white arrow) (Fig. 6C). In addition, we performed an in vitro phosphatase assay by incubating recombinant human DUSP19 with a biotinylated VEGFR3 phosphopeptide. The phospho-signal of the phospho-VEGFR3 peptide was reduced by addition of recombinant DUSP19 (Fig. 6D). Taken together, these results confirmed that DUSP19 directly binds to VEGFR3 and dephosphorylates VEGFR3 via its tyrosine phosphatase activity.

### PTEN loss promotes the invasiveness of pNETs in vivo

**Fig. 7:**
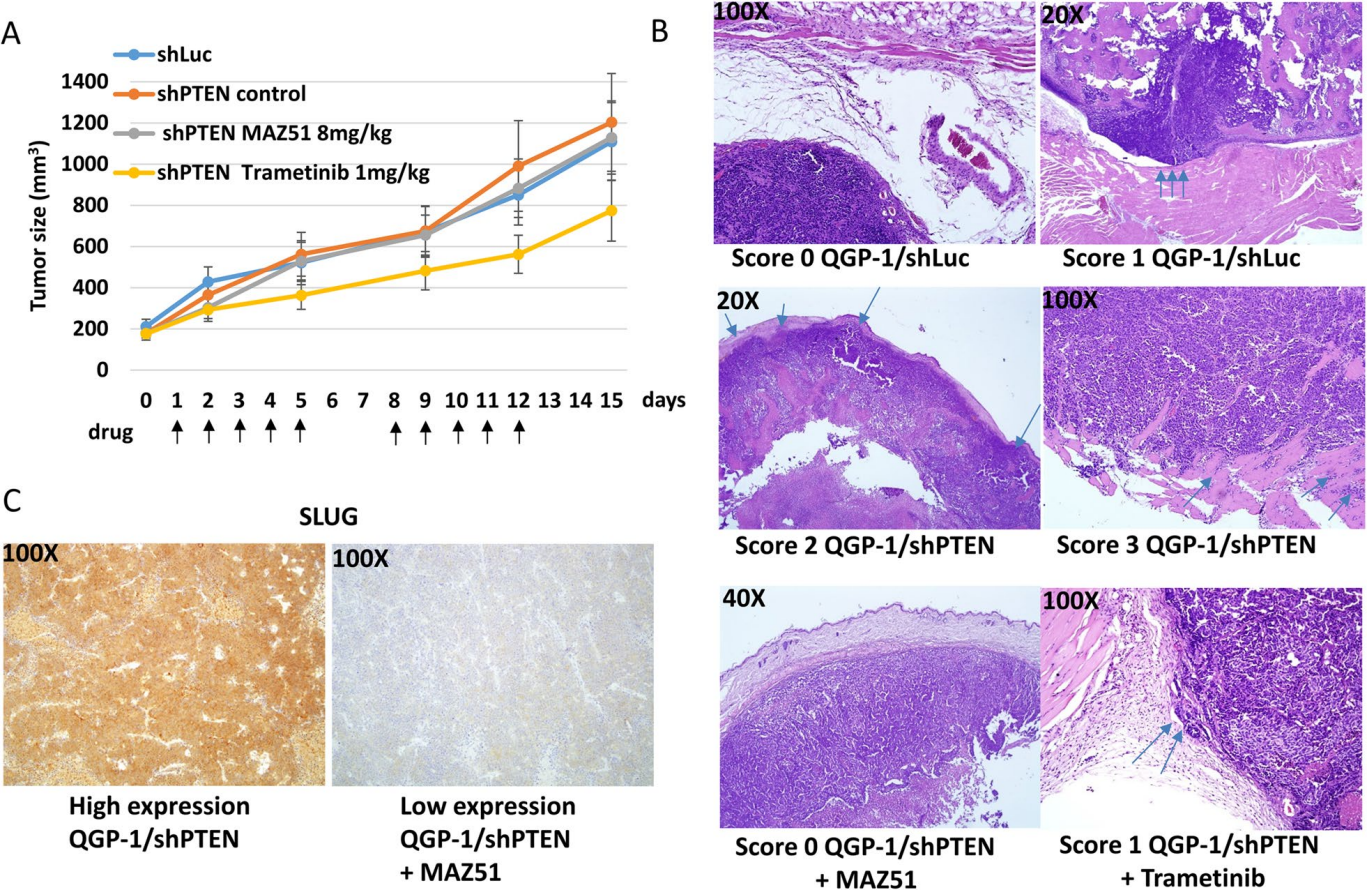
PTEN loss promotes the invasiveness of pNETs in vivo*.* **A** The tumor growth curves of QGP-1 xenograft-bearing mice in the shLuc control, shPTEN control, shPTEN + MAZ51, and shPTEN + trametinib groups. **B** Representative H&E images of QGP-1 xenografts with (shPTEN) and without (shLuc) PTEN loss and treated with MAZ51 (shPTEN + MAZ51) or trametinib (shPTEN + trametinib). Score 0: confinement of the tumor to the original area; score 1: focal invasion into the peripheral fibrofatty tissue; score 2: multifocal invasion into the peripheral fibrofatty tissue; score 3: invasion into muscular bundles. **C** Representative IHC staining of SLUG in xenografts with high (QGP-1/shPTEN) or low (QGP-1/shPTEN + MAZ51) SLUG expression

We performed an animal study to evaluate the effect of PTEN loss on pNET invasiveness in vivo. Tumor growth in QGP-1 xenograft-bearing mice was not significantly increased by PTEN loss (Fig. 7A). We evaluated the invasion status of the tumors by microscopic observation of hematoxylin and eosin (H&E) staining of the excised xenografts and their surrounding tissues. The invasion score was significantly higher in QGP-1 xenografts with PTEN loss than in control tumors (Wilcoxon rank-sum test, *P* = 0.017). Representative H&E-stained images of QGP-1 xenografts with and without PTEN loss are shown in Fig. 7B. We administered vehicle control (PBS), the VEGFR3 inhibitor MAZ51, or the MEK inhibitor trametinib to mice bearing QGP-1 xenografts with PTEN loss for 2 weeks (5 days of drug treatment and 2 days of rest) and evaluated tumor growth and invasiveness by H&E staining. Figure 7A shows that tumor growth was not significantly inhibited by MAZ51 or trametinib. However, tumor invasion in mice bearing QGP-1 xenografts with PTEN loss was significantly suppressed by MAZ51 treatment (Wilcoxon rank-sum test, *P* = 0.021) and tended to be suppressed by trametinib treatment (Wilcoxon rank-sum test, *P* = 0.096). Representative H&E-stained images are shown in Fig. 7B. We evaluated the expression of the EMT marker SLUG in the xenografts. The staining of SLUG was in the cytoplasm of the tumor cells. A SLUG staining score of 3+ in ≥ 30% of the tumors was defined as high SLUG expression. There were five, nine, three, and seven mice with high expression of SLUG in the control (shLuc), shPTEN, shPTEN + MAZ51, and shPTEN + trametinib groups, respectively. The expression of SLUG in xenografts with PTEN loss (shPTEN) tended to be higher than that in the control (shLuc) tumors (Fisher’s exact test, *P* = 0.141), although the difference was not statistically significant. The expression of SLUG in xenografts with PTEN loss was suppressed by MAZ51 treatment (Fisher’s exact test, *P* = 0.020) compared to that in untreated tumors. Figure 7C shows the representative IHC staining of SLUG in the tumors.

## Discussion

In this study, we demonstrated that PTEN loss promotes pNET invasion and migration through the upregulation of VEGFR3 phosphorylation, ERK phosphorylation, and the expression of the EMT molecules vimentin and SLUG in tumor cells. PTEN regulates VEGFR3 phosphorylation through positive regulation of DUSP19 to directly interact with VEGFR3 and dephosphorylate it in pNET cells. The VEGFR3 inhibitor MAZ51 and MEK inhibitor trametinib may reduce the enhanced invasion and migration abilities of pNET cells induced by PTEN loss. The results were validated in a xenograft mouse model.

PTEN is well known for its function as a tumor suppressor and a potent inhibitor of the PI3K-AKT-mTOR pathway to regulate cell growth and survival. PTEN also plays a role in other functions, such as cellular metabolism, motility, polarity and senescence [6]. Aberrant PTEN expression has been shown in various cancer types and is associated with tumor proliferation, metastasis, and prognosis in these cancers [22–24]. Low expression of PTEN has also been shown in pNETs and is associated with shorter patient survival times [7]. Previously, we showed that loss of PTEN and/or LKB1 was associated with increased proliferation of pNET cells [9]. The role of PTEN in the invasive or metastatic capability of pNETs is unknown. In studies regarding the invasive and metastatic capabilities of pNETs, Yang et al. showed that autotaxin and neuropilin-1 are upregulated by STAT3 activation upon IL-6 stimulation and are associated with the metastatic capability of the pNET cell line BON1 [25, 26]. Hunter et al. showed that heparinase, an enzyme that degrades heparan sulfate proteoglycans, promotes peritumoral lymphangiogenesis and tumor invasion in a RIP1-Tag2 (RT2) PanNET transgenic mouse model [27]. Sennino et al. reported that concurrent inhibition of VEGF signaling in the tumor microenvironment and c-MET signaling in tumor cells suppresses pNET growth, invasion, and metastasis [28]. In the current study, we showed that the expression status of PTEN affects pNET invasion and migration.

VEGFR3 has been well reported for its function in tumor invasion and metastasis in addition to lymphangiogenesis [11–14]. Most studies have reported that VEGFR3 signaling in tumor cells depends on the VEGFC/VEGFR3 axis in an autocrine manner and mainly contributes to tumor invasion and metastasis [14, 29, 30]. Matsuura et al. reported that the autocrine loop of VEGFC/FLT-4 (VEGFR3) in tumor cells promotes tumor proliferation and lymphangiogenesis [29]. Yeh et al. showed that VEGFC/VEGFR3 upregulates SLUG expression via the KRAS-MAPK-YAP1 pathway and enhances cancer migration, invasion and stemness [14]. Li et al. demonstrated that OCT4 increases VEGFC expression, which results in VEGFR3 activation and induction of EMT in esophageal cancer. In addition, OCT4/VEGFC/VEGFR3/EMT signaling promoted tumor growth and intraperitoneal metastasis in a xenograft mouse model [30]. Although we previously showed that PTEN loss can upregulate the mTOR/c-Myc axis in pNETs and that c-Myc activation may upregulate VEGFC to promote lymphangiogenesis in pNETs [9, 18]. Our current study showed that the activation of VEGFR3 signaling in pNETs was not abolished by the addition of a VEGFC-neutralizing chimeric protein, R3-Fc, but was blocked by the VEGFR3 inhibitor MAZ51. This result demonstrated that the regulation of VEGFR3 phosphorylation by PTEN is VEGFC-independent.

**Fig. 8:**
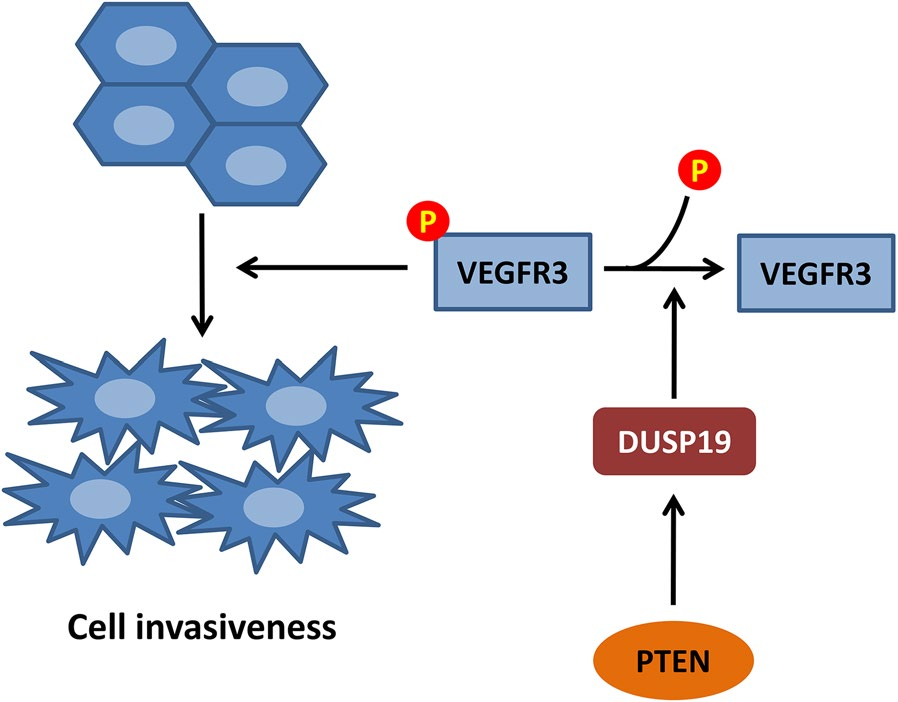
The putative model of the mechanism by which PTEN regulates the invasiveness of pNETs via DUSP19-mediated VEGFR3 dephosphorylation. PTEN positively regulates DUSP19 expression. DUSP19 binds directly to VEGFR3 to dephosphorylate it. Low expression of PTEN induces downregulation of DUSP19 and suppresses DUSP19-mediated dephosphorylation of VEGFR3, which leads to increased VEGFR3 phosphorylation and activation of its downstream ERK signaling pathway and EMT molecules (vimentin and SLUG) to enhance tumor invasiveness

PTEN is a phosphatase that dephosphorylates both lipid and polypeptide substrates [6]. Although the major biological function of PTEN is to dephosphorylate lipid substrates, it has also been reported to dephosphorylate protein substrates, including cAMP-responsive element-binding protein 1 (CREB1) [31] and insulin receptor substrate 1 (IRS1) [19] to perform its tumor-suppressive function. In our study, as PTEN-regulated VEGFR3 phosphorylation in pNETs was independent of VEGFC, we hypothesized that PTEN acts as a protein phosphatase for VEGFR3. However, we failed to demonstrate direct interaction between VEGFR3 and PTEN. Intriguingly, we found that PTEN transcriptionally upregulates DUSP19 to dephosphorylate VEGFR3 and DUSP19 directly interacts with VEGFR3. A weak positive correlation of PTEN and DUSP19 was noted in the RNA sequencing data of 33 pNET tumors although not statistically significant [Spearman’s ρ = 0.1667 (95% CI − 0.187 to 0.482, and *P* = 0.3539)] (Additional file 3: Fig. S3) [17]. Because the sample size was small, further study with larger sample size of pNETs is warranted to confirm the positive correlation of PTEN and DUSP19. DUSP19 is an atypical DUSP that localizes in the cytoplasm. DUSP19 appears to mediate the regulation of MAPK and JNK signaling [21]. Wang et al. showed that DUSP19 expression was reduced in osteoarthritis patients and that DUSP19 inhibited chondrocyte apoptosis via JNK dephosphorylation [32]. Yao et al. showed that DUSP19 is negatively regulated by IL-1β and that overexpression of DUSP19 suppresses the activation of JAK2/STAT3 and matrix metalloproteinase (MMP) expression in rat chondrocytes [33]. Although DUSP19 has been shown to function in the regulation of these signaling pathways, its mechanism of action and its substrate are still unknown. Yao et al. analyzed the receptor tyrosine kinase–protein phosphatase interactome and showed the interaction of DUSP19 with VEGFR3 [20]. In our study, we demonstrated that VEGFR3 is the substrate of DUSP19 and that DUSP19 is positively regulated by PTEN in transcriptional level. These results clarify the association between PTEN, DUSP19, and VEGFR3. In addition, we demonstrated the regulation of the EMT regulator SLUG [34], which has been proven to be a downstream target of VEGFR3 and induces cell invasion [14], by PTEN both in vitro and in vivo. Taken together, our results suggest a novel mechanism for the regulation of invasiveness in pNETs with aberrant PTEN expression (Fig. 8).

In this study, we demonstrated that the activation of VEGFR3/ERK and EMT molecules in pNETs is induced by PTEN loss to promote tumor invasiveness. Therefore, VEGFR3 and ERK can be targeted to inhibit the invasion and metastasis of pNETs. We chose the potent selective VEGFR3 inhibitor MAZ51 and MEK inhibitor trametinib to validate this hypothesis. Our data showed that both MAZ51 and trametinib effectively inhibited PTEN loss-induced increase in pNET invasion and migration. ERK is a downstream effector of VEGFR3 [35, 36]. Trametinib is an MEK inhibitor that inhibits the MEK and ERK signaling pathways and has been approved for use in the treatment of BRAF-mutant cancers, such as melanoma and anaplastic thyroid cancer, in combination with the BRAF inhibitor dabrafenib [37, 38]. It has also been investigated for use in advanced solid tumors in combination with the anti-VEGFR inhibitor pazopanib or chemoradiation in phase I trials [39, 40]. In our study, trametinib inhibited the invasion and migration of pNET cells as effectively as MAZ51 in vitro but less effectively than MAZ51 in vivo via inhibition of the ERK pathway*.* Most VEGFR inhibitors are not VEGFR3 selective. However, new ERK inhibitors and highly selective VEGFR3 inhibitors have been developed and are currently under investigation [41, 42]. The novel agents may be candidate anti-invasive drugs for treating pNETs. Our study identified VEGFR3, MEK, and ERK as potential therapeutic targets for controlling pNET metastasis. However, the animal study did not show significant inhibition of tumor growth. This result suggests that the combination of an anti-invasive agent and an anti-proliferative agent, such as sunitinib, everolimus, or chemotherapy, is worthy of further investigation in the treatment of pNETs. On the other hand, we showed that PTEN regulates VEGFR3 and the downstream signaling pathway of VEGFR3 is mediated through the regulation of DUSP19. Targeting DUSP1 and DUSP6 genetically or with a DSUP inhibitor was shown to inhibit malignant peripheral nerve sheath tumor growth and promote cell death [43]. Novel DUSP inhibitors are under investigation [44, 45]. Therefore, DUSP19 is also a potential therapeutic target for pNETs.

## Conclusions

We demonstrate a novel mechanism by which PTEN loss promotes pNET invasion and migration by downregulating DUSP19 to inhibit DUSP19-mediated VEGFR3 dephosphorylation. Loss of PTEN is commonly observed in pNETs and is associated with poor prognosis. Our results provide important mechanistic clarification of pNET invasiveness and metastasis, and identify VEGFR3, ERK, and DUSP19 as potential therapeutic targets to prevent and treat pNET metastasis.

## Supplementary Information

**Additional file 1: Figure S1.** The migration and invasion abilities of NIT-1 cells are negatively regulated by PTEN. (A) The relative migration (*P* = 0.021, Wilcoxon rank-sum test) and invasion (*P* = 0.021, Wilcoxon rank-sum test) abilities of NIT-1 cells with and without knockdown of PTEN. (B) The protein levels of VEGFR3, phosphorylated VEGFR3, E-cadherin, vimentin and SLUG in NIT-1 cells with and without PTEN knockdown.

**Additional file 2: Figure S2.** VEGFR3 phosphorylation is responsible for pNET cell invasiveness in a VEGFC-independent manner. (A) The relative migration and invasion abilities of NIT-1 cells with (shPTEN) and without (shLuc) knockdown of PTEN and treated with the VEGFR3-Fc chimera protein (shPTEN-R3Fc) or the VEGFR3 inhibitor MAZ51 (shPTEN-MAZ51). Migration: shLuc vs. shPTEN, *P* = 0.021; shPTEN vs. shPTEN-MAZ51, *P* = 0.030; Wilcoxon rank-sum test. Invasion: shLuc vs. shPTEN, *P* = 0.021; shPTEN vs. shPTEN-MAZ51, *P* = 0.030; Wilcoxon rank-sum test. (B) The protein level of phosphorylated VEGFR3 in NIT-1 cells with and without knockdown of PTEN and treated with the VEGFR3-Fc chimera protein R3-Fc or the VEGFR3 inhibitor MAZ51.

**Additional file 3: Figure S3.** DUSP19 showed a weak positive correlation with PTEN in pNET tumors. The mRNA expression of PTEN and DUSP19 from the RNA sequencing data of 33 pancreatic neuroendocrine tumors, which were collected from the GEO GSE118014 database (http://www.ncbi.nlm.nih.gov/geo/). Spearman’s ρ = 0.1667 (95% CI − 0.187 to 0.482, and *P* = 0.3539).

## Data Availability

The datasets used and/or analyzed during the current study are available from the corresponding author on reasonable request.

## Abbreviations

PTEN: Phosphatase and tensin homolog
pNETs: Pancreatic neuroendocrine tumors
VEGFR3: Vascular endothelial growth factor receptor 3
IHC: Immunohistochemical
DUSP: Dual-specificity protein phosphatase
VEGF: Vascular endothelial growth factor
EMT: Epithelial–mesenchymal transition
CREB1: CAMP-responsive element-binding protein 1
IRS1: Insulin receptor substrate 1
PBS: Phosphate-buffered saline
H&E: Hematoxylin and eosin
