# Supplementary figures and images for: PTEN regulates invasiveness in pancreatic neuroendocrine tumors through DUSP19-mediated VEGFR3 dephosphorylation
Source: J Biomed Sci. 2022 Nov 6;29:92. doi: 10.1186/s12929-022-00875-2 (PMC9639322; doi:10.1186/s12929-022-00875-2)

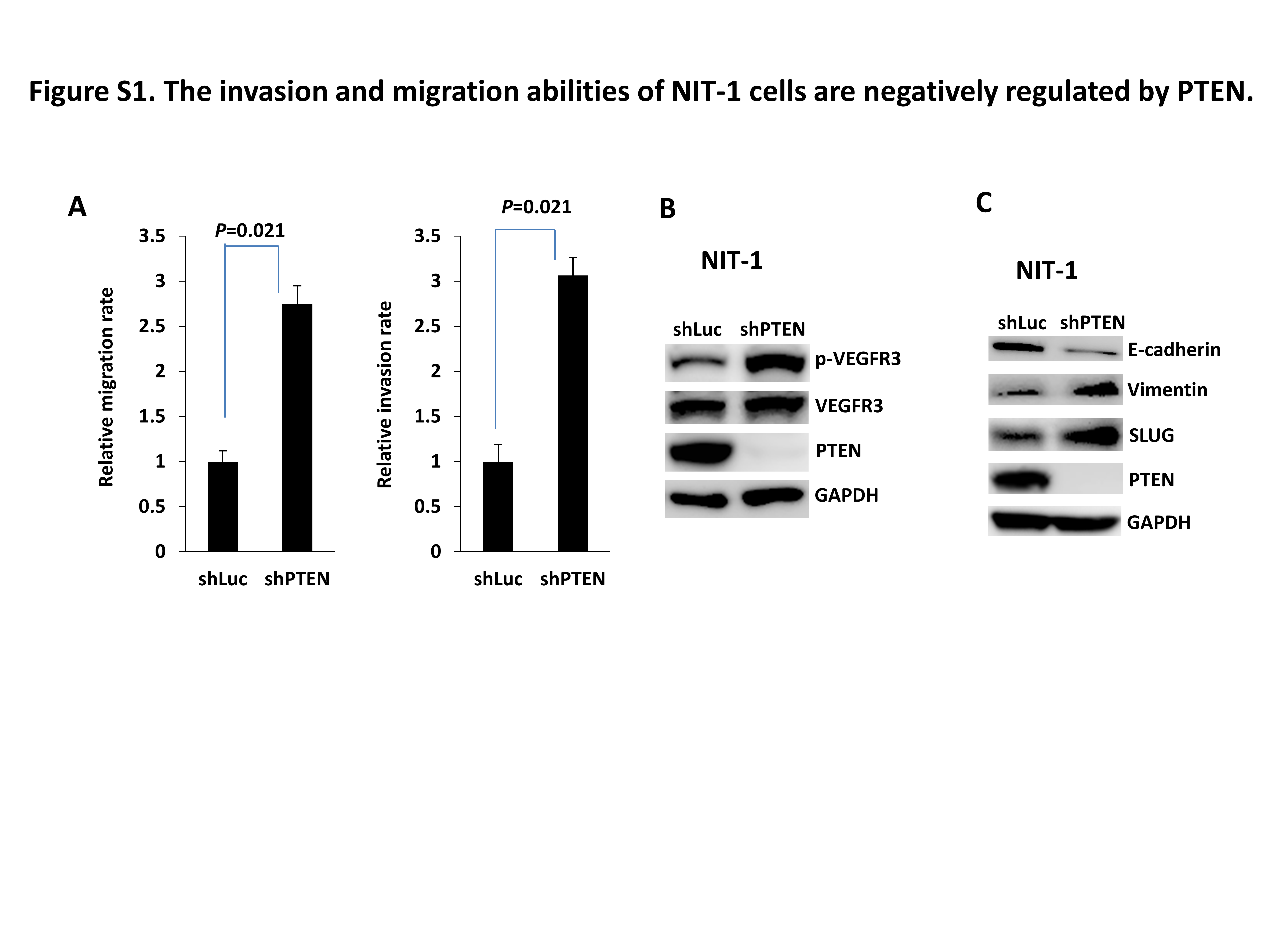

Supplement: Supplementary file 1 — Additional file 1: Figure S1. The migration and invasion abilities of NIT-1 cells are negatively regulated by PTEN. (A) The relative migration (P = 0.021, Wilcoxon rank-sum test) and invasion (P = 0.021, Wilcoxon rank-sum test) abilities of NIT-1 cells with and without knockdown of PTEN. (B) The protein levels of VEGFR3, phosphorylated VEGFR3, E-cadherin, vimentin and SLUG in NIT-1 cells with and without PTEN knockdown. [file 12929_2022_875_MOESM1_ESM.tif]

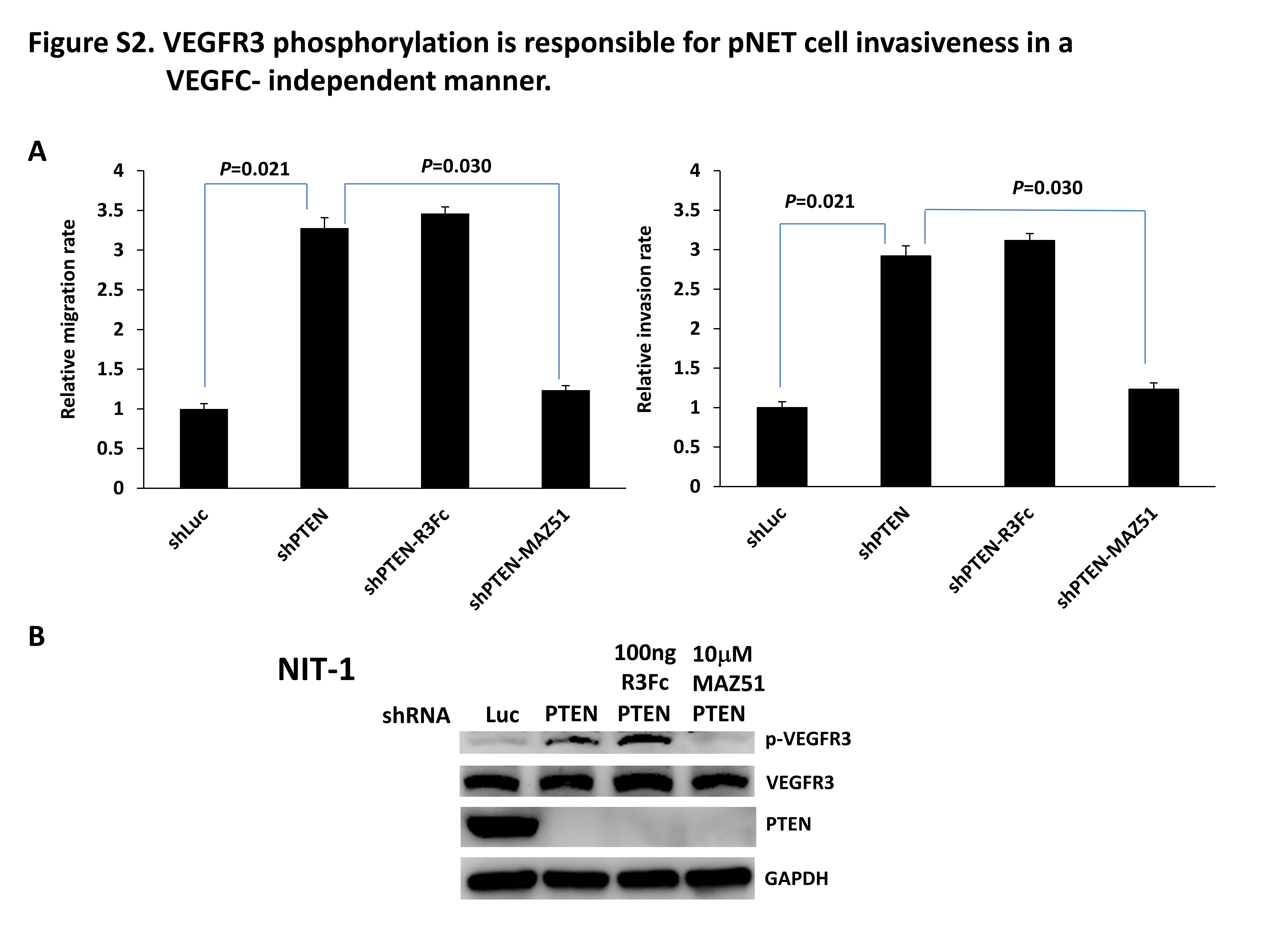

Supplement: Supplementary file 2 — Additional file 2: Figure S2. VEGFR3 phosphorylation is responsible for pNET cell invasiveness in a VEGFC-independent manner. (A) The relative migration and invasion abilities of NIT-1 cells with (shPTEN) and without (shLuc) knockdown of PTEN and treated with the VEGFR3-Fc chimera protein (shPTEN-R3Fc) or the VEGFR3 inhibitor MAZ51 (shPTEN-MAZ51). Migration: shLuc vs. shPTEN, P = 0.021; shPTEN vs. shPTEN-MAZ51, P = 0.030; Wilcoxon rank-sum test. Invasion: shLuc vs. shPTEN, P = 0.021; shPTEN vs. shPTEN-MAZ51, P = 0.030; Wilcoxon rank-sum test. (B) The protein level of phosphorylated VEGFR3 in NIT-1 cells with and without knockdown of PTEN and treated with the VEGFR3-Fc chimera protein R3-Fc or the VEGFR3 inhibitor MAZ51. [file 12929_2022_875_MOESM2_ESM.tif]

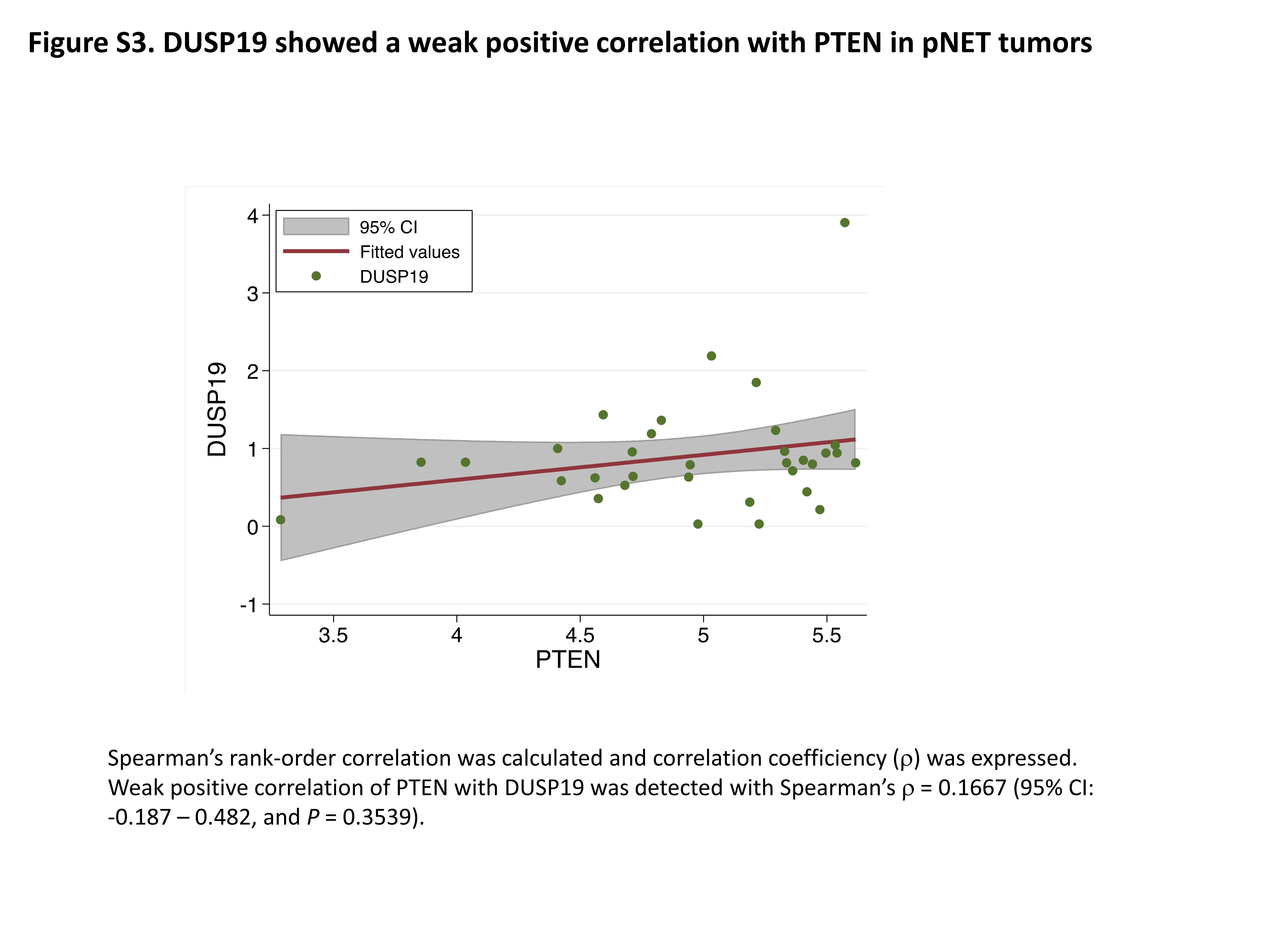

Supplement: Supplementary file 3 — Additional file 3: Figure S3. DUSP19 showed a weak positive correlation with PTEN in pNET tumors. The mRNA expression of PTEN and DUSP19 from the RNA sequencing data of 33 pancreatic neuroendocrine tumors, which were collected from the GEO GSE118014 database (http://www.ncbi.nlm.nih.gov/geo/). Spearman’s ρ = 0.1667 (95% CI − 0.187 to 0.482, and P = 0.3539). [file 12929_2022_875_MOESM3_ESM.tif]
